# Childhood ADHD and autism spectrum disorder difficulties: exploring the impact of copy number variants on young adult outcomes

**DOI:** 10.1192/bjo.2026.11018

**Published:** 2026-04-16

**Authors:** Charlotte A. Dennison, Mia Flanagan, Amy Shakeshaft, Kate Tilling, Lucy Riglin, Anita Thapar

**Affiliations:** Wolfson Centre for Young People’s Mental Health, Cardiff University, UK; Centre for Neuropsychiatric Genetics and Genomics, Division of Psychological Medicine and Clinical Neurosciences, Cardiff University, UK; School of Medicine, Cardiff University, UK; MRC Integrative Epidemiology Unit, Bristol University, UK

**Keywords:** ALSPAC, copy number variant, attention-deficit hyperactivity disorder, autistic spectrum disorder, genetics

## Abstract

Rare copy number variants (CNVs; deleted/duplicated DNA segments) are associated with childhood attention-deficit hyperactivity disorder (ADHD) and autism spectrum disorder (ASD). It is unknown whether carrying a CNV moderates the effect of ADHD/ASD on adult outcomes. In a UK population-based cohort, the Avon Longitudinal Study of Parents and Children, ADHD and ASD difficulties at ages 7–16 years were defined categorically. Outcomes included: General Certificate of Secondary Education non-attainment; depression at ages 18 and 24; functioning at age 25; not in education, employment or training; and receiving state benefits at age 25. Logistic regressions were used to assess associations between ADHD/ASD and outcomes, and to test CNVs as moderators. Multiple imputation was used to account for data missingness. We did not find strong evidence of CNVs moderating the effect of ADHD or ASD on young adult outcomes. However, confidence intervals for the moderating effect were wide, so further research in larger clinical samples is necessary.

Children with neurodevelopmental disorders, including attention-deficit hyperactivity disorder (ADHD) and autism spectrum disorders (ASD), have poorer mental health, social, educational and occupational outcomes than their neurotypical peers.^1,2^ However, there is substantial heterogeneity in outcomes. Copy number variants (CNVs) are deletions and duplications of DNA segments that are enriched in people with neurodevelopmental conditions including ADHD, ASD and intellectual disability.^3^ UK guidelines for psychiatrists recommend routine genetic testing for children with intellectual disability, but not for ADHD or ASD unless there are additional comorbidities, dysmorphic features or unusual medical presentations.^4^ Guidelines in the USA support chromosomal microarray testing for intellectual disability and ASD.^5^ It is currently unknown whether CNV screening of people with ADHD or ASD would help clinicians estimate future outcomes. Using a UK population cohort, we aimed to evaluate whether known neurodevelopmental or large, rare CNVs moderate key ADHD and ASD outcomes.

## Method

Pregnant women residing in Avon, UK and with expected delivery dates between 1 April 1991 and 31 December 1992 were invited to participate in the Avon Longitudinal Study of Parents and Children (ALSPAC). Initially 14 541 mothers were enrolled, with 13 998 children alive at 1 year of age. Following additional recruitment at age 7, the total sample size was 15 447 pregnancies, with 14 901 children alive at age 1 year.^6,7^ We assert that all procedures contributing to this work comply with the ethical standards of the relevant national and institutional committees on human experimentation, and with the Helsinki Declaration of 1975 as revised in 2013. The study and all procedures involving human subjects/patients were approved by the ALSPAC Ethics and Law Committee and the local research ethics committees; our study was approved under project no. B3998. Participants provided written informed consent. Further details on the sample are provided in the Supplementary material available online at https://doi.org/10.1192/bjo.2026.11018.

ADHD was defined as meeting ICD-10 criteria for ADHD at ages 7, 10, 13 or 15 years, assessed via the Development and Well-Being Assessment.^8^ ASD difficulties were defined as a score of >12 (the validated threshold) on the Social Communication Disorders Checklist (SCDC)^9^ at ages 7, 10, 13 or 16 years. Sensitivity analyses were conducted using total scores of the parent-rated Strengths and Difficulties Questionnaire (SDQ)^10^ ADHD subscale and SCDC at age 7.

Outcomes in young adulthood included depression; SDQ impact on functioning; General Certificate of Secondary Education (GCSE) non-attainment; not in education, employment or training (NEET); and being in receipt of state benefits. Depression was defined as meeting ICD-10 criteria for a depressive episode at ages 18 and 24, assessed via the Clinical Interview Schedule–Revised.^11^ Impact on functioning was defined as self-rated difficulties being endorsed as ‘definite’ or ‘severe’ on SDQ at age 25. GCSE non-attainment was defined as self-report of not receiving at least one A*- to C-grade GCSE, asked at age 18. NEET and being in receipt of state benefits at age 25 were defined by self-report.

CNVs were called using PennCNV (version 1.0.5 for Unix; K Wang et al., Philadelphia, PA, USA; https://github.com/WGLab/PennCNV), with filtering applied to include only those meeting the following criteria: (a) log*R* ratio <2.5 s.d. from mean; (b) number of CNVs per individual <100; (c) waviness factor <2.5 s.d. from mean; (d) number of probes ≥20; and (e) confidence score ≥10. Neurodevelopmental CNVs were defined based on published criteria (Supplementary Table 1),^12^ and large, rare CNVs were defined in Plink (version 1.07 for Unix; S Purcell, Boston, MA, USA; https://www.cog-genomics.org/plink/) (Supplementary material).

Logistic regressions assessed associations between exposures (ADHD/ASD) and outcomes, with the presence of a neurodevelopmental CNV included as an interaction with ADHD/ASD, to assess moderation effects. No other covariates were included. Sensitivity analyses first assessed continuous measures of ADHD and ASD traits as exposures and, second, large, rare CNVs as a moderator.

Multiple imputation was performed in R (version 4.4.0 for Windows; R Core Team, Vienna, Austria; https://www.R-project.org/) and is detailed in Supplementary Tables 2 and 3.

## Results

CNV data from 8414 people who passed quality control were included in analyses; 194 people (2.3%) carried a neurodevelopmental CNV and 734 (8.7%) carried any large, rare CNV. Sample sizes prior to imputation are displayed in Supplementary Tables 4 and 5.

ADHD was associated with GCSE non-attainment, depression at age 24, impact on functioning, NEET and receiving state benefits at age 25. There was no evidence of neurodevelopmental CNVs moderating the association between ADHD and depression at age 18, or impact on functioning. Interactions with GCSEs, depression at age 24, NEET and receiving state benefits could not be estimated due to low sample size. Sensitivity analyses of ADHD symptom scores showed the same patterns of association with outcomes, and there was no evidence of associations being moderated by neurodevelopmental CNVs (Table 1). Sensitivity analyses using large, rare CNVs did not show any evidence of moderation effects on outcomes (Supplementary Table 8).

**Table 1:** Association of ADHD or ASD with adult outcomes

Exposure		Main effect				Interaction			
	Outcome	Odds ratio	Lower CI	Upper CI	*P*-value	Odds ratio	Lower CI	Upper CI	*P*-value
ADHD	GCSEs	4.97	2.51	9.83	5.4 × 10^−6^	NA: insufficient sample size			
	Depression T age 18	1.16	0.58	2.33	0.67	2.83	0.25	32.20	0.40
	Depression at age 24	2.75	1.66	4.56	1.0 × 10^−4^	NA: insufficient sample size			
	SDQ impact	3.34	1.93	5.79	2.0 × 10^−5^	1.50	0.07	30.17	0.79
	NEET at age 25	5.70	3.24	10.02	3.5 × 10^−9^	NA: insufficient sample size			
	Receiving state benefits	2.86	1.65	4.95	1.9 × 10^−4^	NA: insufficient sample size			
ASD	GCSEs	3.06	1.90	4.92	5.2 × 10^−6^	0.63	0.05	7.77	0.72
	Depression at age 18	2.25	1.61	3.14	2.2 × 10^−6^	NA: insufficient sample size			
	Depression at age 24	1.84	1.30	2.59	5.5 × 10^−4^	NA: insufficient sample size			
	SDQ impact	2.87	2.03	4.04	4.1 × 10^−9^	NA: insufficient sample size			
	NEET at age 25	4.18	2.80	6.22	9.6 × 10^−12^	NA: insufficient sample size			
	Receiving state benefits	1.90	1.30	2.77	9.2 × 10^−4^	1.38	0.24	8.02	0.72
ADHD traits (continuous)	GCSEs	1.20	1.11	1.29	1.2 × 10^−6^	1.09	0.69	1.72	0.70
	Depression at age 18	1.02	0.97	1.08	0.39	0.91	0.69	1.19	0.49
	Depression at age 24	1.08	1.03	1.13	1.2 × 10^−3^	0.87	0.65	1.15	0.32
	SDQ impact	1.10	1.05	1.15	4.0 × 10^−5^	0.96	0.74	1.24	0.73
	NEET at age 25	1.12	1.05	1.18	4.6 × 10^−4^	0.99	0.70	1.39	0.93
	Receiving state benefits	1.06	1.01	1.12	0.02	1.02	0.79	1.30	0.90
ASD traits (continuous)	GCSEs	1.09	1.05	1.13	6.9 × 10^−8^	0.89	0.63	1.24	0.49
	Depression at age 18	1.06	1.03	1.09	9.7 × 10^−5^	1.04	0.87	1.23	0.68
	Depression at age 24	1.06	1.03	1.10	1.2 × 10^−4^	0.92	0.75	1.14	0.44
	SDQ impact	1.08	1.05	1.12	5.5 × 10^−6^	0.93	0.73	1.19	0.55
	NEET at age 25	1.13	1.09	1.16	8.5 × 10^−13^	0.82	0.55	1.24	0.36
	Receiving state benefits	1.06	1.03	1.09	2.7 × 10^−4^	0.86	0.66	1.13	0.28

Main effect, main association between outcome and exposure; interaction, interaction between neurodevelopmental copy number variants and exposure when predicting each outcome; ADHD, attention-deficit hyperactivity disorder; GCSE, General Certificate of Secondary Education; NA, not available; SDQ, Strengths and Difficulties Questionnaire; NEET, not in education, employment or training; ASD, autism spectrum disorder.

ASD was associated with all outcomes: GCSE non-attainment, depression at 18 and 24 years, impact on functioning, NEET and receiving state benefits at age 25. Neurodevelopmental CNVs did not show evidence of moderating the associations with GCSEs or benefits. Interactions with the remaining outcomes could not be estimated due to low sample size. Sensitivity analyses of ASD symptom scores showed the same pattern of association with outcomes, and did not show evidence of moderation effects by neurodevelopmental CNV carrier status. Sensitivity analyses using large, rare CNVs did not show any evidence of moderation (Supplementary Table 9).

Findings were consistent when analysing complete cases only, as presented in Supplementary Tables 6 and 7.

## Discussion

In a large, UK population cohort, childhood ADHD and ASD were associated with young adult functional and mental health outcomes. However, we did not find evidence to support the presence of moderating effects by neurodevelopmental or large, rare CNVs.

This study was an exploratory investigation of whether routine CNV testing could aid psychiatrists in estimating outcomes, specifically young adult functional outcomes and mental health, in children with broadly defined ADHD or ASD. Evidence from a general adult population suggests that neurodevelopmental CNVs are associated with poorer functional outcomes,^13^ and one study found that CNVs may stratify response to social skills training for people with ASD.^14^ Thus, it is plausible that CNVs may moderate outcomes in ASD/ADHD. However, the impact of CNVs on functional outcomes in ASD or ADHD, to the best of our knowledge, has not been evaluated. Our findings do not provide evidence of strong moderating effects of CNVs in people with ASD and ADHD, and therefore do not support their utility in estimating outcomes in clinical settings. Instead, our findings suggest that the role of CNVs in ASD/ADHD may be limited to aetiology. However, confidence intervals for moderating effects were wide, and included effects sufficiently large to be clinically significant or effects too small for clinical significance. Thus we recommend replicating this analysis in well-powered samples, in order to improve confidence in our findings and make well-informed clinical decisions. Further research is needed to assess CNV moderation effects at different ages, because the findings may be different.

We relied on questionnaire-based assessments of ASD rather than diagnosis, although research suggests that the enrichment of CNVs extends to ASD and ADHD traits captured by these assessments.^15^ Although we used multiple imputation, sample attrition is a further limitation, meaning that some moderation effects could not be analysed due to lack of data. CNVs are rare, so power to detect moderation was low.

Overall, our study shows that, within the limitations of existing data, there is no strong evidence that CNVs have a large moderating effect on the association between ADHD or ASD and functional outcomes or depression in young adulthood.

## Supporting information

10.1192/bjo.2026.11018.sm001 Dennison et al. supplementary material Dennison et al. supplementary material

## Data Availability

ALSPAC data are owned by the University of Bristol and are accessible to bona fide researchers. Researchers can apply for access via the University of Bristol website: https://www.bristol.ac.uk/alspac/researchers/access/.

## Supplementary material

The supplementary material is available online at https://doi.org/10.1192/bjo.2026.11018
