# Supplementary material for: Childhood ADHD and autism spectrum disorder difficulties: exploring the impact of copy number variants on young adult outcomes
Source: BJPsych Open. 2026 Apr 16;12(3):e108. doi: 10.1192/bjo.2026.11018 (PMC13107328; doi:10.1192/bjo.2026.11018)
Supplement: Dennison et al. supplementary material [file S2056472426110187sup001.docx]

Table of Contents

[Supplementary methods 1](#_Toc213932409)

[*ALSPAC* 1](#_Toc213932410)

[*CNV Calling* 2](#_Toc213932411)

[Table S1 Criteria used to define neurodevelopmental CNVs. 3](#_Toc213932412)

[*Multiple imputation* 6](#_Toc213932413)

[Table S2. Variables imputed via multiple imputation, and the variables used to impute them. 6](#_Toc213932414)

[Table S3. Proportion of missing data for each item in the model. 8](#_Toc213932415)

[Supplementary Results 9](#_Toc213932416)

[Table S4. Sample sizes prior to imputation, stratified by large CNV carrier status. 9](#_Toc213932417)

[Table S5. Sample sizes prior to imputation, stratified by ND CNV carrier status. 10](#_Toc213932418)

[Table S6. Association of ADHD or ASD and each adult outcome in complete cases. 11](#_Toc213932419)

[Table S7. Interaction between large rare CNVs and ADHD or ASD in complete cases 12](#_Toc213932420)

[Table S8. Interaction between ADHD and large rare CNVs. 13](#_Toc213932421)

[Table S9. Interaction between ASD and large rare CNVs. 14](#_Toc213932422)

[References 15](#_Toc213932423)

[STROBE Statement 16](#_Toc213932424)

## Supplementary methods

### *ALSPAC*

For details of data collected on ALSPAC index children since the age of 18 years, please see Northstone et al.^1^. Study data were collected and managed using REDCap electronic data capture tools hosted at the University of Bristol^2^. REDCap (Research Electronic Data Capture) is a secure, web-based software platform designed to support data capture for research studies. Please note that the study website contains details of all the data that is available through a fully searchable data dictionary and variable search tool http://www.bristol.ac.uk/alspac/researchers/our-data/. Consent for biological samples has been collected in accordance with the Human Tissue Act (2004). Informed consent for the use of data collected via questionnaires and clinics was obtained from participants following the recommendations of the ALSPAC Ethics and Law Committee at the time. Further information on ethical approval of individual aspects of ALSPAC data collection can be found here: <https://www.bristol.ac.uk/alspac/researchers/research-ethics/>.

### *CNV Calling*

CNV calling was performed in accordance with the Cardiff MRC Pathfinder pipeline (<https://github.com/CardiffMRCPathfinder/NeurodevelopmentalCNVCalling.git>). First, SNPs were filtered to exclude on the basis of: 1) Hardy-Weinberg equilibrium p<1x10^-4^, 2) genotyping rate <0.95, and 3) minor allele frequency <0.01. Next, PennCNV was used to call CNVs, and adjacent CNV calls were merged if the distance between them was <50% of the combined length of the CNVs.

We focus on known neurodevelopmental CNVs^3,4^ as these have been widely researched in the literature and a largely agreed upon to have the strongest evidence of association with neurodevelopmental disorders^5–7^. However, we acknowledge that this is unlikely to be an exhaustive list of all CNVs that could influence ASD/ADHD and therefore also evaluate any rare CNVs to capture a wider range of variation, consistent with previous literature^8^.

### Table S1 Criteria used to define neurodevelopmental CNVs.

Criteria from Kendall et al. 2017^4^

| CNV | Criteria |
| --- | --- |
| 1p36 del (GABRD) | Size >50% of critical region, affecting GABRD |
| 1p36 dup (GABRD) | Size >50% of critical region, affecting GABRD |
| TAR del 1q21.2 | Size >50% of critical region |
| TAR dup 1q21.1 | Size >50% of critical region |
| 1q21.1 del | Size >50% of critical region |
| 1q21.1 dup | Size >50% of critical region |
| NRXN1 del 2p16.3 | Exonic deletions |
| 2q11.2 del (LMAN2L, ARID5A) | Size >50% of critical region, affecting both LMAN2L and ARID5A |
| 2q13 del | Size >50% of critical region |
| 2q13 dup | Size >50% of critical region |
| 2q37 del (HDAC4) | Size >50% of critical region, affecting HDAC4 |
| 3q29 del | Size >50% of critical region |
| Wolf-Hirschhorn del (4p16.3) | Size >50% of critical region |
| Wolf-Hirschhorn dup (4p16.3) | Size >50% of critical region |
| Sotos syndrome del (5q35) | Size >50% of critical region |
| Williams-Beuren syndrome del (7q11.23) | Size >50% of critical region |
| Williams-Beuren syndrome dup (7q11.23) | Size >50% of critical region |
| 8p23.1 del | At least 1Mbp of critical region |
| 8p23.1 dup | At least 1Mbp of critical region |
| 9q34 del (EHMT1) | At least 1Mbp CNVs, including EHMT1 |
| 10q23 del (NRG3, GRID1) | At least 1Mbp, including NRG3 and GRID1 |
| Potocki-Shaffer syndrome del (EXT2) (11p11.2) | Size >50% of critical region, including EXT2 |
| 15q11.2 del BP1-BP2 | Size >50% of critical region PWS del/dup Full critical region, ~4Mbp |
| 15q11.2 dup BP1-BP2 | Size >50% of critical region PWS del/dup Full critical region, ~4Mbp |
| Prader-Willi syndrome/Angelman syndrome del (15q11.2q12) | Full critical region, ~4Mbp |
| Prader-Willi syndrome/Angelman syndrome dup (15q11.2q12) | Full critical region, ~4Mbp |
| 15q13.3 del BP4-BP5 | Size >50% of critical region |
| 15q24 del | At least 1Mbp between the A‐E intervals |
| 15q24 dup | At least 1Mbp between the A‐E intervals |
| 15q25 del | At least 1Mbp between the A‐D intervals |
| 16p13.11 del | Size >50% of critical region |
| 16p13.11 dup | Size >50% of critical region |
| 16p12.1 del | Size >50% of critical region |
| 16p11.2 distal del | Size >50% of critical region |
| 16p11.2 distal dup | Size >50% of critical region |
| 16p11.2 del | Size >50% of critical region |
| 16p11.2 dup | Size >50% of critical region |
| 17p13.3 del (YWHAE) | Exonic deletions; whole gene duplications |
| 17p13.3 dup (YWHAE) | Exonic deletions; whole gene duplications |
| 17p13.3 del (PAFAH1B1) | Exonic deletions; whole gene duplications |
| 17p13.3 dup (PAFAH1B1) | Exonic deletions; whole gene duplications |
| Potocki-Lupski syndrome dup (17p11.2) | Size >50% of critical region |
| Smith-Magenis syndrome del (17p11.2) | Size >50% of critical region |
| 17q11.2 del (NF1) | Size >50% of critical region, affecting NF1 |
| 17q11.2 dup (NF1) | Size >50% of critical region, affecting NF1 |
| Renal cysts and diabetes syndrome del (17q12) | Size >50% of critical region |
| 17q12 dup | Size >50% of critical region |
| 17q21.31 del | Size >50% of critical region |
| 22q11.2 del | Size >50% of critical region |
| 22q11.2 dup | Size >50% of critical region |
| 22q11.2 distal del | Size >50% of critical region |
| 22q11.2 distal dup | Size >50% of critical region |
| SHANK3 del (22q13) | At least 1Mbp CNVs, including SHANK3 |
| SHANK3 dup (22q13) | At least 1Mbp CNVs, including SHANK3 |

### *Multiple imputation*

The multiple imputation model included all variables in the primary and sensitivity analyses, as well as known predictors of missingness, which included: maternal age at birth, maternal education, parity, smoking in pregnancy, birthweight, gestational age, and home ownership status^9^. A two-stage procedure was performed to identify the minimum number of imputed datasets required to ensure stable effect sizes and standard errors^10^, which recommended a minimum of 198 datasets. 200 datasets were imputed in R using the package ‘mice’ and the predictive mean matching method. Imputation was performed separately based on CNV carrier status, to allow for accurate imputation and analysis of the interaction effect^11^. Table S2 details which variables were included to impute each variable in the analysis. Genetic data was not imputed and only participants with high quality genetic data were included. Table S2 reports the proportion of missing data for each item included in the imputation model.

### Table S2. Variables imputed via multiple imputation, and the variables used to impute them.

| **Variable being imputed** | **Variables used to impute** | | | |
| --- | --- | --- | --- | --- |
|  | **Predictors of missingness** | **Outcome variables** | **Neurodevelopmental variables** | **Genetic variables** |
| ADHD (binary) | Maternal age, maternal education, parity, smoking in pregnancy, birthweight, gestational age, home ownership | GCSE non-attainment, depression age 18, depression age 24, SDQ impact, NEET age 25, receiving state benefits age 25 | SDQ hyperactivity total age 7, 9, and 13 | Large rare CNV, ND CNV |
| ASD (binary) | Maternal age, maternal education, parity, smoking in pregnancy, birthweight, gestational age, home ownership | GCSE non-attainment, depression age 18, depression age 24, SDQ impact, NEET age 25, receiving state benefits age 25 | SCDC total age 7, 10, and 13 | Large rare CNV, ND CNV |
| SDQ hyperactivity age 7 | Maternal age, maternal education, parity, smoking in pregnancy, birthweight, gestational age, home ownership | GCSE non-attainment, depression age 18, depression age 24, SDQ impact, NEET age 25, receiving state benefits age 25 | ADHD (binary), SDQ hyperactivity total age 9 and 13 | Large rare CNV, ND CNV |
| SCDC score age 7 | Maternal age, maternal education, parity, smoking in pregnancy, birthweight, gestational age, home ownership | GCSE non-attainment, depression age 18, depression age 24, SDQ impact, NEET age 25, receiving state benefits age 25 | ASD (binary), SCDC total age 10 and 13 | Large rare CNV, ND CNV |
| GCSE non-attainment | Maternal age, maternal education, parity, smoking in pregnancy, birthweight, gestational age, home ownership | Depression age 18, depression age 24, SDQ impact, NEET age 25, receiving state benefits age 25 | ADHD (binary), ASD (binary), SDQ total age 7, SCDC total age 7 | Large rare CNV, ND CNV |
| Depression age 18 | Maternal age, maternal education, parity, smoking in pregnancy, birthweight, gestational age, home ownership | GCSE non-attainment, depression age 24, SDQ impact, NEET age 25, receiving state benefits age 25 | ADHD (binary), ASD (binary), SDQ total age 7, SCDC total age 7 | Large rare CNV, ND CNV |
| Depression age 24 | Maternal age, maternal education, parity, smoking in pregnancy, birthweight, gestational age, home ownership | GCSE non-attainment, depression age 18, SDQ impact, NEET age 25, receiving state benefits age 25 | ADHD (binary), ASD (binary), SDQ total age 7, SCDC total age 7 | Large rare CNV, ND CNV |
| SDQ impact | Maternal age, maternal education, parity, smoking in pregnancy, birthweight, gestational age, home ownership | GCSE non-attainment, depression age 18, depression age 24, NEET age 25, receiving state benefits age 25 | ADHD (binary), ASD (binary), SDQ total age 7, SCDC total age 7 | Large rare CNV, ND CNV |
| NEET age 25 | Maternal age, maternal education, parity, smoking in pregnancy, birthweight, gestational age, home ownership | GCSE non-attainment, depression age 18, depression age 24, SDQ impact, receiving state benefits age 25 | ADHD (binary), ASD (binary), SDQ total age 7, SCDC total age 7 | Large rare CNV, ND CNV |
| Receiving state benefits age 25 | Maternal age, maternal education, parity, smoking in pregnancy, birthweight, gestational age, home ownership | GCSE non-attainment, depression age 18, depression age 24, SDQ impact, NEET age 25 | ADHD (binary), ASD (binary), SDQ total age 7, SCDC total age 7 | Large rare CNV, ND CNV |

### Table S3. Proportion of missing data for each item in the model.

Only individuals with high-quality genetic data were included (n=8,414).

| **Variable** | **Role** | **Percentage with missing data** |
| --- | --- | --- |
| SDQ hyperactivity age 7 | Exposure | 32.6% |
| SCDC score age 7 | Exposure | 33.6% |
| ADHD (binary) | Exposure | 21.0% |
| ASD (binary) | Exposure | 21.3% |
| SDQ impact | Outcome | 63.2% |
| Depression age 18 | Outcome | 59.5% |
| Depression age 24 | Outcome | 65.4% |
| GCSE non-attainment | Outcome | 70.8% |
| NEET age 25 | Outcome | 65.8% |
| Receiving state benefits age 25 | Outcome | 65.2% |
| Large rare CNV | Exposure | 0.0% |
| ND CNV | Exposure | 0.0% |
| Maternal age | Auxiliary | 5.2% |
| Maternal education | Auxiliary | 11.9% |
| Parity | Auxiliary | 10.1% |
| Smoking in pregnancy | Auxiliary | 8.6% |
| Birthweight | Auxiliary | 6.3% |
| Gestational age | Auxiliary | 5.0% |
| Home ownership | Auxiliary | 12.5% |

## Supplementary Results

### Table S4. Sample sizes prior to imputation, stratified by large CNV carrier status.

| **Outcome** | **Outcome status** | **CNV status** | **ADHD** | | **ASD** | |
| --- | --- | --- | --- | --- | --- | --- |
|  |  |  | **No** | **Yes** | **No** | **Yes** |
| GCSE | Attained GCSE | No large CNV | 2076 | 33 | 1970 | 137 |
|  |  | Large CNV | 181 | <5 | 172 | 11 |
|  | No GCSE | No large CNV | 67 | 7 | 58 | 15 |
|  |  | Large CNV | 6 | 0 | 6 | 0 |
| Depression 18 | No depression at 18 | No large CNV | 2654 | 56 | 2511 | 194 |
|  |  | Large CNV | 224 | 5 | 212 | 17 |
|  | Depression at 18 | No large CNV | 259 | 5 | 226 | 38 |
|  |  | Large CNV | 20 | <5 | 19 | <5 |
| Depression 24 | No depression at 24 | No large CNV | 2224 | 40 | 2126 | 135 |
|  |  | Large CNV | 186 | 7 | 177 | 16 |
|  | Depression at 24 | No large CNV | 264 | 11 | 242 | 31 |
|  |  | Large CNV | 25 | 0 | 24 | <5 |
| Impact on functioning | No impact on functioning | No large CNV | 2342 | 27 | 2231 | 131 |
|  |  | Large CNV | 202 | <5 | 195 | 11 |
|  | Impact on functioning | No large CNV | 286 | 14 | 255 | 43 |
|  |  | Large CNV | 34 | <5 | 29 | 6 |
| NEET | Not NEET at 25 | No large CNV | 2323 | 30 | 2202 | 144 |
|  |  | Large CNV | 206 | <5 | 198 | 11 |
|  | NEET at 25 | No large CNV | 129 | 9 | 114 | 23 |
|  |  | Large CNV | 12 | <5 | 11 | <5 |
| Receiving state benefits | Not receiving benefits | No large CNV | 2265 | 30 | 2146 | 143 |
|  |  | Large CNV | 197 | <5 | 191 | 10 |
|  | Receiving benefits | No large CNV | 222 | 9 | 205 | 24 |
|  |  | Large CNV | 24 | <5 | 21 | <5 |

### Table S5. Sample sizes prior to imputation, stratified by ND CNV carrier status.

| **Outcome** | **Outcome status** | **CNV status** | **ADHD** | | **ASD** | |
| --- | --- | --- | --- | --- | --- | --- |
|  |  |  | **No** | **Yes** | **No** | **Yes** |
| GCSE | Attained GCSE | No ND CNV | 2221 | 34 | 2108 | 145 |
|  |  | ND CNV | 36 | <5 | 34 | <5 |
|  | No GCSE | No ND CNV | 71 | 7 | 63 | 14 |
|  |  | ND CNV | <5 | 0 | <5 | <5 |
| Depression 18 | No depression at 18 | No ND CNV | 2823 | 58 | 2675 | 202 |
|  |  | ND CNV | 55 | <5 | 48 | 9 |
|  | Depression at 18 | No ND CNV | 272 | 6 | 237 | 41 |
|  |  | ND CNV | 7 | <5 | 8 | 0 |
| Depression 24 | No depression at 24 | No ND CNV | 2370 | 43 | 2267 | 144 |
|  |  | ND CNV | 40 | <5 | 36 | 7 |
|  | Depression at 24 | No ND CNV | 283 | 11 | 260 | 32 |
|  |  | ND CNV | 6 | 0 | 6 | 0 |
| Impact on functioning | No impact on functioning | No ND CNV | 2503 | 30 | 2390 | 137 |
|  |  | ND CNV | 41 | <5 | 36 | 5 |
|  | Impact on functioning | No ND CNV | 314 | 14 | 277 | 49 |
|  |  | ND CNV | 6 | <5 | 7 | 0 |
| NEET | Not NEET at 25 | No ND CNV | 2489 | 31 | 2364 | 150 |
|  |  | ND CNV | 40 | <5 | 36 | 5 |
|  | NEET at 25 | No ND CNV | 138 | 11 | 122 | 26 |
|  |  | ND CNV | <5 | 0 | <5 | 0 |
| Receiving state benefits | Not receiving benefits | No ND CNV | 2427 | 32 | 2303 | 151 |
|  |  | ND CNV | 35 | <5 | 34 | <5 |
|  | Receiving benefits | No ND CNV | 237 | 10 | 220 | 25 |
|  |  | ND CNV | 9 | 0 | 6 | <5 |

### Table S6. Association of ADHD or ASD and each adult outcome in complete cases.

Columns refer to Odds Ratio (OR), lower and upper 95% confidence intervals (CI), and the p-value of i) the main association between the outcome and the exposure, and ii) the interaction between ND CNVs and the exposure when predicting each outcome.

| **Exposure** |  | **Main effect** | | | | **Interaction** | | | |
| --- | --- | --- | --- | --- | --- | --- | --- | --- | --- |
|  | **Outcome** | **OR** | **Lower CI** | **Upper CI** | **Pvalue** | **OR** | **Lower CI** | **Upper CI** | **Pvalue** |
| ADHD | GCSEs | 6.18 | 2.66 | 14.39 | 2.3E-05 | NA - insufficient sample size | | | |
|  | Depression age 18 | 1.18 | 0.54 | 2.61 | 0.68 | 2.44 | 0.19 | 31.00 | 0.49 |
|  | Depression age 24 | 1.95 | 1.00 | 3.81 | 0.05 | NA - insufficient sample size | | | |
|  | SDQ Impact | 3.85 | 2.05 | 7.20 | 2.6E-05 | 1.84 | 0.09 | 35.88 | 0.69 |
|  | NEET at 25 | 5.98 | 2.96 | 12.08 | 6.2E-07 | NA - insufficient sample size | | | |
|  | Receiving state benefit | 2.94 | 1.44 | 6.03 | 3.2E-03 | NA - insufficient sample size | | | |
| ASD | GCSEs | 3.39 | 1.89 | 6.10 | 4.5E-05 | 3.51 | 0.16 | 75.76 | 0.42 |
|  | Depression age 18 | 2.16 | 1.51 | 3.09 | 2.6E-05 | NA - insufficient sample size | | | |
|  | Depression age 24 | 1.83 | 1.23 | 2.74 | 3.1E-03 | NA - insufficient sample size | | | |
|  | SDQ Impact | 2.95 | 2.08 | 4.17 | 1.0E-09 | NA - insufficient sample size | | | |
|  | NEET at 25 | 3.22 | 2.05 | 5.06 | 4.1E-07 | NA - insufficient sample size | | | |
|  | Receiving state benefit | 1.89 | 1.24 | 2.90 | 3.3E-03 | 4.90 | 0.64 | 37.64 | 0.13 |
| ADHD traits (continuous) | GCSEs | 1.30 | 1.18 | 1.43 | 7.9E-08 | NA - insufficient sample size | | | |
|  | Depression age 18 | 1.03 | 0.97 | 1.09 | 0.30 | 0.87 | 0.59 | 1.28 | 0.47 |
|  | Depression age 24 | 1.07 | 1.01 | 1.13 | 0.02 | 0.74 | 0.47 | 1.16 | 0.19 |
|  | SDQ Impact | 1.10 | 1.04 | 1.16 | 5.1E-04 | 0.98 | 0.72 | 1.34 | 0.89 |
|  | NEET at 25 | 1.11 | 1.03 | 1.19 | 0.01 | 0.96 | 0.57 | 1.64 | 0.89 |
|  | Receiving state benefit | 1.05 | 0.98 | 1.11 | 0.15 | 1.01 | 0.73 | 1.41 | 0.95 |
| ASD traits (continuous) | GCSEs | 1.12 | 1.06 | 1.19 | 5.4E-05 | 1.07 | 0.59 | 1.94 | 0.83 |
|  | Depression age 18 | 1.06 | 1.02 | 1.09 | 1.3E-03 | 1.07 | 0.84 | 1.37 | 0.56 |
|  | Depression age 24 | 1.06 | 1.03 | 1.10 | 8.8E-04 | 0.75 | 0.44 | 1.27 | 0.28 |
|  | SDQ Impact | 1.08 | 1.04 | 1.11 | 2.7E-05 | 0.83 | 0.55 | 1.25 | 0.37 |
|  | NEET at 25 | 1.15 | 1.10 | 1.20 | 2.4E-11 | 0.57 | 0.12 | 2.66 | 0.48 |
|  | Receiving state benefit | 1.05 | 1.01 | 1.10 | 0.01 | 0.61 | 0.30 | 1.25 | 0.18 |

### Table S7. Interaction between large rare CNVs and ADHD or ASD in complete cases

Columns refer to the Odds ratio (OR), lower 95% confidence interval (CI), upper 95% CI, and p-value of the interaction between ADHD and large rare CNVs, when predicting each outcome.

| **Exposure** | **Outcome** | **Interaction** | | | |
| --- | --- | --- | --- | --- | --- |
|  |  | **OR** | **Lower CI** | **Upper CI** | **Pvalue** |
| ADHD (binary) | GCSEs | NA - insufficient sample size | | | |
|  | Depression age 18 | 4.90 | 0.71 | 33.97 | 0.11 |
|  | Depression age 24 | NA - insufficient sample size | | | |
|  | SDQ impact | 0.35 | 0.03 | 3.55 | 0.37 |
|  | NEET at 25 | 2.12 | 0.28 | 16.15 | 0.47 |
|  | Receiving state benefits | 0.67 | 0.06 | 7.08 | 0.74 |
| ASD (binary) | GCSEs | NA - insufficient sample size | | | |
|  | Depression age 18 | 0.90 | 0.23 | 3.55 | 0.89 |
|  | Depression age 24 | 0.23 | 0.03 | 1.88 | 0.17 |
|  | SDQ impact | 1.28 | 0.41 | 3.95 | 0.67 |
|  | NEET at 25 | 1.59 | 0.36 | 7.08 | 0.54 |
|  | Receiving state benefits | 2.07 | 0.55 | 7.79 | 0.28 |
| ADHD traits (continuous) | GCSEs | 0.81 | 0.55 | 1.19 | 0.29 |
|  | Depression age 18 | 0.97 | 0.78 | 1.20 | 0.78 |
|  | Depression age 24 | 0.97 | 0.80 | 1.18 | 0.75 |
|  | SDQ impact | 0.95 | 0.81 | 1.12 | 0.57 |
|  | NEET at 25 | 1.24 | 0.96 | 1.60 | 0.10 |
|  | Receiving state benefits | 0.98 | 0.80 | 1.20 | 0.84 |
| ASD traits (continuous) | GCSEs | 0.88 | 0.64 | 1.21 | 0.43 |
|  | Depression age 18 | 0.95 | 0.83 | 1.09 | 0.46 |
|  | Depression age 24 | 0.90 | 0.77 | 1.05 | 0.19 |
|  | SDQ impact | 0.92 | 0.81 | 1.05 | 0.24 |
|  | NEET at 25 | 1.07 | 0.92 | 1.23 | 0.39 |
|  | Receiving state benefits | 0.93 | 0.79 | 1.10 | 0.41 |

### Table S8. Interaction between ADHD and large rare CNVs.

Results of interaction analysis of imputed data measuring the association and interaction between i) ADHD (binary item) and ii) ADHD traits (continuous score) and large rare CNV carrier status for each outcome.

Columns refer to the Odds ratio (OR), lower 95% confidence interval (CI), upper 95% CI, and p-value of the interaction between ADHD and large rare CNVs, when predicting each outcome.

| **Exposure** | **Outcome** | **Interaction** | | | |
| --- | --- | --- | --- | --- | --- |
|  |  | **OR** | **Lower CI** | **Upper CI** | **Pvalue** |
| ADHD (binary) | GCSEs | NA - insufficient sample size | | | |
|  | Depression age 18 | 3.66 | 0.55 | 24.19 | 0.18 |
|  | Depression age 24 | NA - insufficient sample size | | | |
|  | SDQ impact | 0.36 | 0.03 | 3.73 | 0.39 |
|  | NEET at 25 | 3.43 | 0.59 | 19.94 | 0.17 |
|  | Receiving state benefits | 1.10 | 0.12 | 9.96 | 0.93 |
| ADHD traits (continuous) | GCSEs | 0.89 | 0.68 | 1.17 | 0.41 |
|  | Depression age 18 | 0.98 | 0.81 | 1.18 | 0.80 |
|  | Depression age 24 | 0.97 | 0.82 | 1.15 | 0.72 |
|  | SDQ impact | 0.99 | 0.85 | 1.14 | 0.87 |
|  | NEET at 25 | 1.16 | 0.95 | 1.41 | 0.14 |
|  | Receiving state benefits | 0.98 | 0.82 | 1.17 | 0.81 |

### Table S9. Interaction between ASD and large rare CNVs.

Results of interaction analysis of imputed data measuring the association and interaction between i) ASD (binary item) and ii) ASD traits (continuous score) and large rare CNV carrier status for each outcome.

Columns refer to the Odds ratio (OR), lower 95% confidence interval (CI), upper 95% CI, and p-value of the interaction between ADHD and large rare CNVs, when predicting each outcome.

| **Exposure** | **Outcome** | **Interaction** | | | |
| --- | --- | --- | --- | --- | --- |
|  |  | **OR** | **Lower CI** | **Upper CI** | **Pvalue** |
| ASD (binary) | GCSEs | NA - insufficient sample size | | | |
|  | Depression age 18 | 1.03 | 0.30 | 3.60 | 0.96 |
|  | Depression age 24 | 0.56 | 0.12 | 2.56 | 0.45 |
|  | SDQ impact | 1.27 | 0.44 | 3.72 | 0.66 |
|  | NEET at 25 | 1.71 | 0.51 | 5.72 | 0.39 |
|  | Receiving state benefits | 1.43 | 0.39 | 5.25 | 0.59 |
| ASD traits (continuous) | GCSEs | 0.93 | 0.75 | 1.15 | 0.51 |
|  | Depression age 18 | 0.96 | 0.85 | 1.09 | 0.52 |
|  | Depression age 24 | 0.93 | 0.83 | 1.06 | 0.28 |
|  | SDQ impact | 0.94 | 0.84 | 1.04 | 0.23 |
|  | NEET at 25 | 1.06 | 0.95 | 1.17 | 0.30 |
|  | Receiving state benefits | 0.94 | 0.81 | 1.08 | 0.36 |

## References

1 Northstone K, Lewcock M, Groom A, Boyd A, Macleod J, Timpson NJ, *et al.* The Avon Longitudinal Study of Parents and Children (ALSPAC): an update on the enrolled sample of index children in 2019. *Wellcome Open Research* 2019. (https://wellcomeopenresearch.org/articles/4-51/v1).

2 Harris PA, Taylor R, Thielke R, Payne J, Gonzalez N, Conde JG. Research electronic data capture (REDCap)--a metadata-driven methodology and workflow process for providing translational research informatics support. *J Biomed Inform* 2009; **42**: 377–81.

3 Coe BP, Witherspoon K, Rosenfeld JA, van Bon BWM, Vulto-van Silfhout AT, Bosco P, *et al.* Refining analyses of copy number variation identifies specific genes associated with developmental delay. *Nat Genet* 2014; **46**: 1063–71.

4 Kendall KM, Rees E, Escott-Price V, Einon M, Thomas R, Hewitt J, *et al.* Cognitive Performance Among Carriers of Pathogenic Copy Number Variants: Analysis of 152,000 UK Biobank Subjects. *Biological Psychiatry* 2017; **82**: 103–10.

5 Birnbaum R, Mahjani B, Loos RJF, Sharp AJ. Clinical Characterization of Copy Number Variants Associated With Neurodevelopmental Disorders in a Large-scale Multiancestry Biobank. *JAMA Psychiatry* 2022; **79**: 250–9.

6 Dennison CA, Martin J, Shakeshaft A, Riglin L, Powell V, Kirov G, *et al.* Early manifestations of neurodevelopmental copy number variants in children: A population-based investigation. *Biological Psychiatry* 2025. doi:10.1016/j.biopsych.2025.03.004.

7 Martin CL, Wain KE, Oetjens MT, Tolwinski K, Palen E, Hare-Harris A, *et al.* Identification of Neuropsychiatric Copy Number Variants in a Health Care System Population. *JAMA Psychiatry* 2020; **77**: 1276–85.

8 Martin J, Tammimies K, Karlsson R, Lu Y, Larsson H, Lichtenstein P, *et al.* Copy number variation and neuropsychiatric problems in females and males in the general population. *American Journal of Medical Genetics Part B: Neuropsychiatric Genetics* 2019; **180**: 341–50.

9 Riglin L, Wootton RE, Thapar AK, Livingston LA, Langley K, Collishaw S, *et al.* Variable Emergence of Autism Spectrum Disorder Symptoms From Childhood to Early Adulthood. *AJP* 2021; **178**: 752–60.

10 von Hippel PT. How Many Imputations Do You Need? A Two-stage Calculation Using a Quadratic Rule. *Sociological Methods & Research* 2020; **49**: 699–718.

11 Bartlett J. Multiple imputation with interactions and non-linear terms. The Stats Geek. 2014. (https://thestatsgeek.com/2014/05/10/multiple-imputation-with-interactions-and-non-linear-terms/).

## STROBE Statement

|  | Item No | Recommendation | Complete |
| --- | --- | --- | --- |
| **Title and abstract** | 1 | (*a*) Indicate the study’s design with a commonly used term in the title or the abstract | X |
|  |  | (*b*) Provide in the abstract an informative and balanced summary of what was done and what was found | X |
| Introduction | | |  |
| Background/rationale | 2 | Explain the scientific background and rationale for the investigation being reported | X |
| Objectives | 3 | State specific objectives, including any prespecified hypotheses | X |
| Methods | | |  |
| Study design | 4 | Present key elements of study design early in the paper | X |
| Setting | 5 | Describe the setting, locations, and relevant dates, including periods of recruitment, exposure, follow-up, and data collection | X |
| Participants | 6 | (*a*) *Cohort study*—Give the eligibility criteria, and the sources and methods of selection of participants. Describe methods of follow-up  *Case-control study*—Give the eligibility criteria, and the sources and methods of case ascertainment and control selection. Give the rationale for the choice of cases and controls  *Cross-sectional study*—Give the eligibility criteria, and the sources and methods of selection of participants | X |
|  |  | (*b*) *Cohort study*—For matched studies, give matching criteria and number of exposed and unexposed  *Case-control study*—For matched studies, give matching criteria and the number of controls per case | X |
| Variables | 7 | Clearly define all outcomes, exposures, predictors, potential confounders, and effect modifiers. Give diagnostic criteria, if applicable | X |
| Data sources/ measurement | 8* | For each variable of interest, give sources of data and details of methods of assessment (measurement). Describe comparability of assessment methods if there is more than one group | *X* |
| Bias | 9 | Describe any efforts to address potential sources of bias | X |
| Study size | 10 | Explain how the study size was arrived at | X |
| Quantitative variables | 11 | Explain how quantitative variables were handled in the analyses. If applicable, describe which groupings were chosen and why | X |
| Statistical methods | 12 | (*a*) Describe all statistical methods, including those used to control for confounding | X |
|  |  | (*b*) Describe any methods used to examine subgroups and interactions | X |
|  |  | (*c*) Explain how missing data were addressed | X |
|  |  | (*d*) *Cohort study*—If applicable, explain how loss to follow-up was addressed  *Case-control study*—If applicable, explain how matching of cases and controls was addressed  *Cross-sectional study*—If applicable, describe analytical methods taking account of sampling strategy | X |
|  |  | (*e*) Describe any sensitivity analyses | X |

| Results | | | Complete |
| --- | --- | --- | --- |
| Participants | 13* | (a) Report numbers of individuals at each stage of study—eg numbers potentially eligible, examined for eligibility, confirmed eligible, included in the study, completing follow-up, and analysed | X |
|  |  | (b) Give reasons for non-participation at each stage | X |
|  |  | (c) Consider use of a flow diagram | X |
| Descriptive data | 14* | (a) Give characteristics of study participants (eg demographic, clinical, social) and information on exposures and potential confounders | X |
|  |  | (b) Indicate number of participants with missing data for each variable of interest | X |
|  |  | (c) *Cohort study*—Summarise follow-up time (eg, average and total amount) | X |
| Outcome data | 15* | *Cohort study*—Report numbers of outcome events or summary measures over time |  |
|  |  | *Case-control study—*Report numbers in each exposure category, or summary measures of exposure | X |
|  |  | *Cross-sectional study—*Report numbers of outcome events or summary measures |  |
| Main results | 16 | (*a*) Give unadjusted estimates and, if applicable, confounder-adjusted estimates and their precision (eg, 95% confidence interval). Make clear which confounders were adjusted for and why they were included | X |
|  |  | (*b*) Report category boundaries when continuous variables were categorized | X |
|  |  | (*c*) If relevant, consider translating estimates of relative risk into absolute risk for a meaningful time period |  |
| Other analyses | 17 | Report other analyses done—eg analyses of subgroups and interactions, and sensitivity analyses | X |
| Discussion | | |  |
| Key results | 18 | Summarise key results with reference to study objectives | X |
| Limitations | 19 | Discuss limitations of the study, taking into account sources of potential bias or imprecision. Discuss both direction and magnitude of any potential bias | X |
| Interpretation | 20 | Give a cautious overall interpretation of results considering objectives, limitations, multiplicity of analyses, results from similar studies, and other relevant evidence | X |
| Generalisability | 21 | Discuss the generalisability (external validity) of the study results | X |
| Other information | | |  |
| Funding | 22 | Give the source of funding and the role of the funders for the present study and, if applicable, for the original study on which the present article is based | X |
